# Risk factors for and prediction of post-intubation hypotension in critically ill adults: A multicenter prospective cohort study

**DOI:** 10.1371/journal.pone.0233852

**Published:** 2020-08-31

**Authors:** Nathan J. Smischney, Rahul Kashyap, Ashish K. Khanna, Ernesto Brauer, Lee E. Morrow, Mohamed O. Seisa, Darrell R. Schroeder, Daniel A. Diedrich, Ashley Montgomery, Pablo Moreno Franco, Uchenna R. Ofoma, David A. Kaufman, Ayan Sen, Cynthia Callahan, Chakradhar Venkata, Gozde Demiralp, Rudy Tedja, Sarah Lee, Mariya Geube, Santhi I. Kumar, Peter Morris, Vikas Bansal, Salim Surani, On behalf of SCCM Discovery (Critical Care Research Network of Critical Care Medicine) HEMAIR Investigators Consortium

**Affiliations:** 1 Department of Anesthesiology and Perioperative Medicine, Mayo Clinic, Rochester, Minnesota, United States of America; 2 HEModynamic and AIRway Management (HEMAIR) Study Group, Mayo Clinic, Rochester, Minnesota, United States of America; 3 Outcomes Research Consortium, Cleveland Clinic, Cleveland, Ohio, United States of America; 4 Department of Anesthesia, Section on Critical Care Medicine, Wake Forest School of Medicine, Winston-Salem, North Carolina, United States of America; 5 Department of Critical Care Medicine, Aurora Health Care, Milwaukee, Wisconsin, United States of America; 6 Department of Critical Care Medicine, Creighton University, Omaha, Nebraska, United States of America; 7 Department of Biostatistics, Mayo Clinic, Rochester, Minnesota, United States of America; 8 Department of Anesthesia and Critical Care Medicine, University of Kentucky, Lexington, Kentucky, United States of America; 9 Department of Critical Care Medicine, Mayo Clinic, Jacksonville, Florida, United States of America; 10 Division of Critical Care Medicine, Geisinger Health System, Danville, Pennsylvania, United States of America; 11 Section of Pulmonary, Critical Care, and Sleep Medicine, Bridgeport Hospital/Yale New Haven Health, Bridgeport, Connecticut, United States of America; 12 Department of Critical Care Medicine, Mayo Clinic, Scottsdale, Arizona, United States of America; 13 Department of Critical Care Medicine, Berkshire Medical Center, Pittsfield, Massachusetts, United States of America; 14 Department of Critical Care Medicine, Mercy Hospital, St. Louis, Missouri, United States of America; 15 Department of Anesthesia and Critical Care Medicine, University of Oklahoma Health Sciences Center, Oklahoma City, Oklahoma, United States of America; 16 Department of Critical Care Medicine, Memorial Medical Center, Modesto, California, United States of America; 17 Division of Pulmonary, Critical Care & Sleep Medicine, Detroit Medical Center, Detroit, Michigan, United States of America; 18 Department of Critical Care Medicine, Kerk School University of Southern California, Los Angeles, California, United States of America; 19 Department of Critical Care Medicine, Corpus Christi Medical Center, Corpus Christi, Texas, United States of America; National Yang-Ming University, TAIWAN

## Abstract

**Objective:**

Hypotension following endotracheal intubation in the ICU is associated with poor outcomes. There is no formal prediction tool to help estimate the onset of this hemodynamic compromise. Our objective was to derive and validate a prediction model for immediate hypotension following endotracheal intubation.

**Methods:**

A multicenter, prospective, cohort study enrolling 934 adults who underwent endotracheal intubation across 16 medical/surgical ICUs in the United States from July 2015-January 2017 was conducted to derive and validate a prediction model for immediate hypotension following endotracheal intubation. We defined hypotension as: 1) mean arterial pressure <65 mmHg; 2) systolic blood pressure <80 mmHg and/or decrease in systolic blood pressure of 40% from baseline; 3) or the initiation or increase in any vasopressor in the 30 minutes following endotracheal intubation.

**Results:**

Post-intubation hypotension developed in 344 (36.8%) patients. In the full cohort, 11 variables were independently associated with hypotension: increasing illness severity; increasing age; sepsis diagnosis; endotracheal intubation in the setting of cardiac arrest, mean arterial pressure <65 mmHg, and acute respiratory failure; diuretic use 24 hours preceding endotracheal intubation; decreasing systolic blood pressure from 130 mmHg; catecholamine and phenylephrine use immediately prior to endotracheal intubation; and use of etomidate during endotracheal intubation. A model excluding unstable patients’ pre-intubation (those receiving catecholamine vasopressors and/or who were intubated in the setting of cardiac arrest) was also developed and included the above variables with the exception of sepsis and etomidate. In the full cohort, the 11 variable model had a C-statistic of 0.75 (95% CI 0.72, 0.78). In the stable cohort, the 7 variable model C-statistic was 0.71 (95% CI 0.67, 0.75). In both cohorts, a clinical risk score was developed stratifying patients’ risk of hypotension.

**Conclusions:**

A novel multivariable risk score predicted post-intubation hypotension with accuracy in both unstable and stable critically ill patients.

**Study registration:**

Clinicaltrials.gov identifier: NCT02508948 and Registered Report Identifier: RR2-10.2196/11101.

## Introduction

Hypotension in critically ill patients in the intensive care unit (ICU) is associated with an increasing risk of myocardial injury, mortality, and acute kidney injury (AKI). Recent work has seen that for every unit increase in time weighted average of mean arterial pressure (MAP) <65 mmHg, the odds of in-hospital mortality increased 11.4% and the odds of AKI increased 7.0% [1]. Further, exposure to any duration of hypotension at a MAP <75 mmHg nearly doubles the odds of myocardial injury and mortality in the ICU [2].

Respiratory failure requiring endotracheal intubation (ETI) occurs commonly [3]. Unfortunately, this life-saving procedure is frequently complicated with significant hypotension and hypoxia [4]. Post-intubation hypotension (PIH) has been recognized as a likely contributor to unfavorable patient outcomes [5–7]. Almost a third of trauma patients who are hypotensive post-intubation suffer mortality [5]. Nearly half of a cohort of 479 critically ill patients experienced PIH, which was associated with significant increases in overall mortality, ICU length of stay, duration of mechanical ventilation, and need for renal replacement therapy [6].

Certain risk factors are known to be associated with immediate hypotension surrounding ETIs. These including, increasing age, higher illness severity, ETI for acute respiratory failure, emergent ETI, use of paralytics, and pre-existing renal failure and chronic obstructive pulmonary disease have all been associated with PIH [7–9]. Intubation medications have been implicated as possible risk factors for PIH based on their mechanism of action (i.e. decreased systemic vascular resistance). However, this risk is not universally recognized and there is substantial variance in best practices associated with safer ETI processes in the ICU [6, 9, 10–17]. Further, despite the above cited associations, there is no currently available formal method that predicts the onset of hypotension when an airway is established in the ICU. Therefore, hypotensive events in this population often happen acutely, and in a rather unexpected fashion, which makes for sub-optimal responses and inadequate proactive preparation.

Given the lack of predictability and harm associated with PIH in critically ill patients, the HEModynamic and AIRway (HEMAIR) study aimed to examine the current state of ETI in ICUs throughout the United States to derive and validate a prediction model for immediate hypotension in this environment.

## Materials and methods

### Institutional approval

The HEMAIR study was approved by the Institutional Review Boards at participating centers (Mayo Clinic Rochester, Scottsdale, and Jacksonville Institutional Review Boards; Cleveland Clinic Institutional Review Board; Aurora Health Care Institutional Review Board; Creighton University Institutional Review Board; University of Kentucky Institutional Review Board; Geisinger Health System Institutional Review Board; Yale New Haven Health Institutional Review Board; Berkshire Medical Center Institutional Review Board; Mercy Hospital Institutional Review Board; University of Oklahoma Institutional Review Board; Memorial Medical Center Institutional Review Board; Detroit Medical Center Institutional Review Board; Kerk School University of Southern California Institutional Review Board; Corpus Christi Medical Center Institutional Review Board), with Mayo Clinic Rochester Institutional Review Board serving as the primary regulatory body. The study was conducted under a waiver of consent. All sites were responsible for entering ETI data at their institutions. The study was registered at Clinicaltrials.gov (identifier-NCT02508948) (Registered Report Identifier RR2-10.2196/11101).

### Study population and protocol

The current study was a prospective, multicenter, cohort study of critically ill adult (≥18 years) patients who underwent ETI in 16 ICUs throughout the United States from July 2015 to January 2017. The study included general, cardiac, and trauma surgery ICUs and medical ICU patients with various diagnoses including neurological patients across 7 Health & Human Services (HHS) regions in the United States. Endotracheal intubations performed outside the ICU, centers with ≤5 enrollments, and patients with unavailable pre/post- blood pressure data were excluded.

A pre-specified standardized case report form, developed by anesthesia, medicine, and pulmonary critical care physicians, focused on two periprocedural aspects of the ETI process—hemodynamic and airway management [18]. The various sites entered data into the Research Electronic Data Capture platform, which was managed and analyzed at Mayo Clinic Rochester. Data were obtained for the hospital stay with emphasis on 60 minutes pre- and 60 minutes post-intubation. Each site collected the data which were then verified by study site personnel with data quality checks performed at study conclusion. To assist with data collection and standardization, a registry (https://www.haemair.com/) and monthly HEMAIR investigator meetings were established. Regarding airway management, rapid sequence intubation was defined *a priori* [18]. This was a pragmatic study and as such the ETI process was not standardized. However, data were collected prospectively during and following ETI. Data elements were pre-specified. A standardized operating manual was established to assist study sites.

Our primary outcome was PIH, defined as: any MAP <65 mmHg; any systolic blood pressure (SBP) <80 mm Hg or a decrease in SBP of 40% from baseline; or the initiation, or increase in infusion rate of any vasoactive agent in the 30-minute window following ETI [5–7]. Baseline blood pressure was defined using the value recorded 15 minutes prior to ETI. If this blood pressure assessment was missing, then the value up to 30 minutes prior to ETI was used as the baseline.

### Sample-size and statistical analysis

The sample-size of the initial cohort was determined for the aim of estimating the overall incidence of PIH and hypoxemia/difficult airway with precision of approximately ±1% [N = 804]. The analysis cohort for the present study included patients who had blood pressure measurements available at baseline and 15 minutes post-intubation. As a secondary analysis, we excluded patients who were receiving pre-intubation catecholamine vasopressors and/or who were intubated in the setting of cardiac arrest to arrive at a cohort of stable patients. Candidate predictor variables were selected *a priori* based on literature review (S2 Table) [6–9, 19–21]. Data for the candidate predictor variables are presented separately for the full and stable cohorts using mean±standard deviation or median (interquartile range) for continuous variables, and frequency counts (percentages) for categorical variables. Data were available for >98% of patients for all candidate predictor variables with the exception of SpO_2_, fluid balance, Acute Physiologic And Chronic Health Evaluation (APACHE) II score (24 hours prior to ETI) and lactate which were missing for 6%, 12%, 16% and 29% respectively. Missing data for lactate was thought to potentially be missing not at random and therefore this variable was dropped from the list of candidate variables. For model building, a single dataset was created which had complete data for all candidate predictor variables. For this dataset, missing data were imputed with SAS, PROC MI using Fully Conditional Specification methods.

Due to the large number of candidate variables considered, modeling was performed utilizing Least Absolute Shrinkage and Selection Operator penalized logistic regression with the penalty parameter (λ) chosen using 10-fold cross-validation. The Box-Tidwell test and supplemental graphical displays were used to assess modeling assumptions for continuous predictor variables. Based on these analyses, baseline SBP was modeled using a linear term representing mmHg below 130 mmHg with a value of zero assigned to those with baseline SBP ≥130 mmHg, and baseline MAP was modeled using a linear term for mmHg below 95 mmHg with a value of zero assigned to those with baseline MAP ≥95 mmHg.

We made the decision *a priori* to perform model building with baseline SBP as the only blood pressure measure included as a candidate predictor. A sensitivity analysis was conducted that included MAP instead of SBP as a candidate predictor variable. The regression coefficients for the variables selected for inclusion into the 2 final models were then obtained from the full and stable cohorts utilizing Least Absolute Shrinkage and Selection Operator penalized logistic regression.

Model discrimination was assessed using the C-statistic and calibration was assessed using graphical displays of the observed and expected percentage of patients experiencing hypotension. To facilitate clinical usefulness, a risk score was created for both the full and stable cohort models [22]. All analyses were performed using SAS version 9.4 (SAS Institute Inc.) and R statistical software version 3.4.1 (R Foundation for Statistical Computing).

## Results

### Cohort characteristics

**Fig 1:**
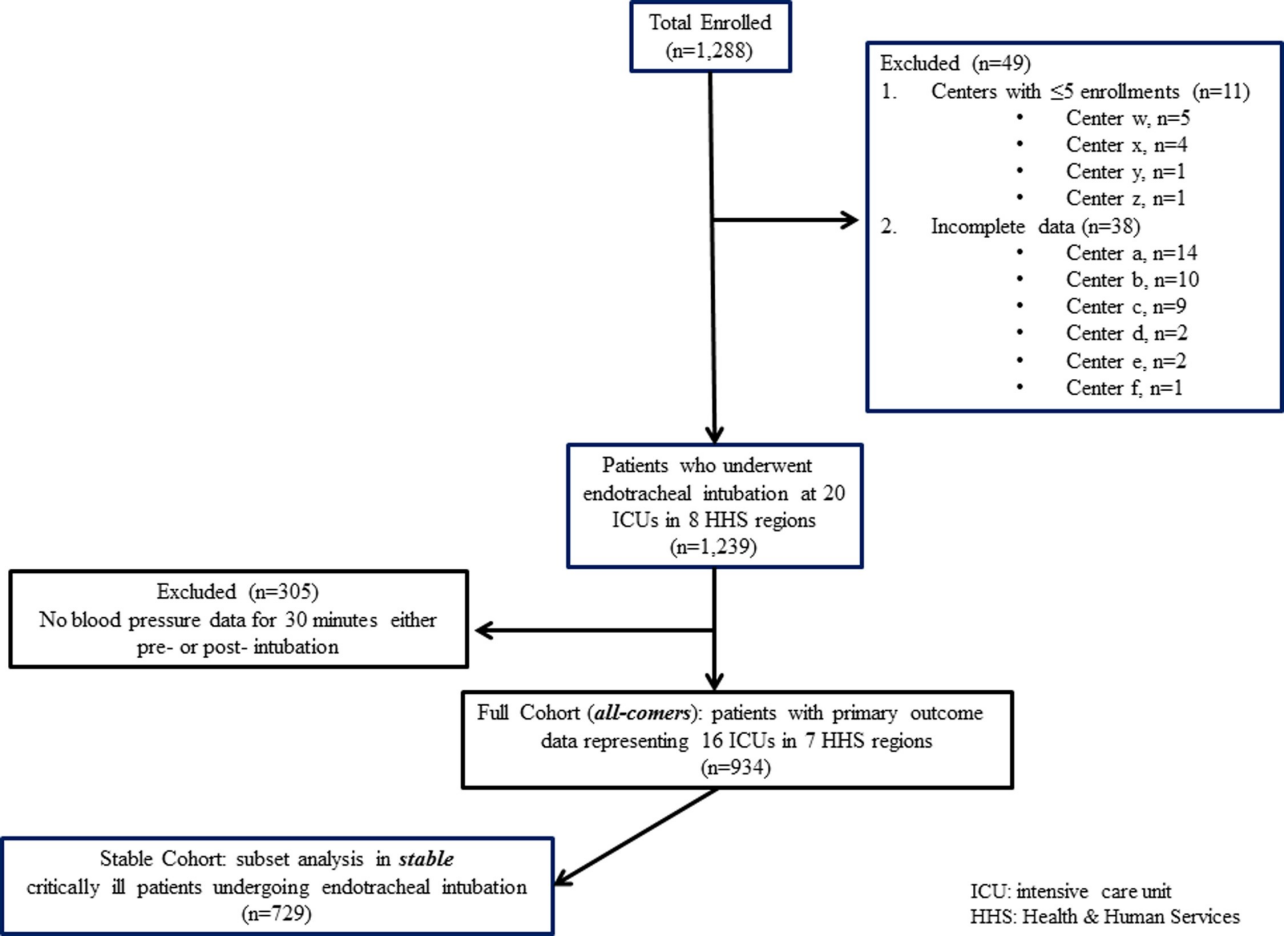
Participant flow diagram. ICU: intensive care unit, HHS: Health & Human Services.

From the original 1,288 patients, 354 were excluded due to incomplete data, centers with ≤5 enrollments, and missing pre- or post-intubation blood pressure data. Thus, the final study population included 934 patients from 16 centers representing 7 HHS regions with729 patients in the stable cohort (Fig 1 and S3 Table). The mean age was 62.4±15.6 years in the full cohort and 61.6±15.8 years in the stable cohort. Most participants were male in both cohorts: 534 (57.2%) in the full set and 419 (57.5%) in the stable set. The majority of patients in both cohorts (Full: 686 [73.5%] and Stable: 539 [73.9%]) were intubated for acute respiratory failure, with 508 (54.4%) patients in the full and 377 (51.7%) patients in the stable cohorts undergoing emergent ETIs (ETI without delay) (Table 1).

**Table 1:** Patient characteristics.

Characteristic		Full Cohort N = 934*	Stable Cohort N = 729^†^
Age (years), mean ± SD		62.4±15.6	61.6±15.8
Sex, n (%)			
	Male	534 (57.2)	419 (57.5)
	Female	400 (43.8)	310 (42.5)
Body mass index, kg/m^2^		29.4±9.1	29.1±8.6
Medical history, n (%)			
	Previous difficult endotracheal intubation	13 (1.4)	12 (1.7)
	Congestive heart failure	198 (21.2)	129 (17.7)
	Coronary artery disease	244 (26.1)	185 (25.4)
	Obstructive lung disease	199 (21.3)	171 (23.5)
	End-stage renal disease	72 (7.7)	50 (6.7)
	Cirrhosis	105 (11.2)	74 (10.2)
	Diabetes mellitus type 2	278 (29.8)	215 (29.5)
	Acute kidney injury stage 1 or higher (AKIN or RIFLE)	322 (34.5)	232 (31.8)
	Dialysis or renal replacement therapy	64 (6.9)	39 (5.4)
	Mechanical circulatory support (VAD, IABP, ECMO)	19 (2.0)	11 (1.5)
	Sepsis-3 (2016)	386 (41.3)	272 (37.3)
	Hypovolemic shock^b^	123 (13.2)	63 (8.6)
Emergency intubation, n (%)		508 (54.4)	377 (51.7)
Intubation setting, n (%)			
	Airway protection	600 (64.2)	462 (63.4)
	Acute respiratory failure	686 (73.5)	539 (73.9)
	Neurologic	217 (23.2)	181 (24.8)
	Cardiac arrest	39 (4.2)	0 (0.0)
	MAP < 65 mmHg—hemodynamic decompensation	175 (18.7)	78 (10.7)
	Procedural-related	146 (15.6)	122 (16.7)

*Data were available for > 98% of patients for all characteristics listed.

^†^Hypovolemic shock: critical decrease in intravascular volume leading to inadequate perfusion (as measured by decreased urine output or increased lactate) resulting in imbalance between oxygen supply/demand.

SD: standard deviation; AKIN: acute kidney injury network; RIFLE: risk, injury, failure, loss of kidney function and end-stage kidney disease; VAD: ventricular assist device; IABP: intra-aortic balloon pump; ECMO: extracorporeal membrane oxygenation; MAP: mean arterial pressure.

The mean baseline systolic / diastolic blood pressure was 123.8±31.8 mmHg / 68.6±21.3 mmHg in the full cohort and 128.6±30.2 mmHg / 71.1±21.4 mmHg in the stable cohort. Four-hundred and sixty-seven (50%) patients in the full and 385 (52.8%) patients in the stable cohorts were intubated by a trainee (fellow, resident, medical student). Etomidate was the most used sedative for ETI (Full: 504 [54%] vs. Stable: 402 [55.1%]) (Table 2).

**Table 2:** Peri-intubation characteristics.

Characteristic			Full Cohort N = 934*	Stable Cohort N = 729*
24 hours prior to endotracheal intubation				
	APACHE II score, mean ± SD		17.5±8.3	16.7±8.1
	Cardiovascular medications, n (%)			
		Diuretics	154 (16.5)	119 (16.3)
		Alpha blocker	11 (1.2)	8 (1.1)
		Beta blocker	97 (10.4)	83 (11.4)
		Ace inhibitors	3 (0.3)	3 (0.4)
		Midodrine	14 (1.5)	7 (1.0)
		Calcium channel blocker	63 (6.8)	57 (7.8)
		Nitrates	28 (3.0)	23 (3.2)
		Anti-arrhythmic	90 (9.6)	64 (8.9)
	RBC transfusion, n (%)		111 (11.9)	74 (10.2)
	Non-RBC transfusion, n (%)		63 (6.8)	44 (6.0)
	Fluid balance (ml), mean ± SD		+522±2153	+224±1867
	Non-invasive ventilation		282 (30.2)	223 (30.6)
	High flow nasal cannula		36 (3.9)	31 (4.3)
	Sedative/hypnotic medication (benzodiazepines, ketamine, opioids, dexmedetomidine)		267 (28.6)	218 (29.9)
	Fluid bolus (≥ 500 ml of crystalloid or colloid)		152 (16.3)	81 (11.1)
	Vasopressors			
		Calcium	13 (1.4)	4 (0.6)
		Catecholamine	179 (19.2)	0 (0.0)
		Vasopressin	45 (4.8)	0 (0.0)
		Phenylephrine	51 (5.5)	34 (4.7)
		Inotrope (dobutamine or milrinone)	19 (2.0)	7 (1.0)
	Hemoglobin (g/dL) mean ± SD		10.1±2.5	10.2±2.5
	Lactate (mmol/L), median (IQR)		1.6 (0.8, 3.1)	1.5 (0.8, 2.7)
	SpO_2_ (%), mean ± SD		94.7±6.5	94.8±6.1
	Systolic blood pressure (mmHg), mean ± SD		123.8±31.3	128.6±30.2
	Diastolic blood pressure (mmHg), mean ± SD		68.6±21.3	71.1±21.4
	Mean arterial pressure (mmHg), mean ± SD		87.0±22.5	90.3±22.0
	Shock index, mean ± SD		0.87±0.29	0.84±0.25
	Modified shock index, mean ± SD		1.24±0.40	1.19±0.36
	Consultant/attending		370 (39.6)	264 (36.2)
	Mid-level (CRNA, NP/PA, RRT)		97 (10.4)	80 (11.0)
	Trainee (fellow, resident, medical student)		467 (50.0)	385 (52.8)
	Ketamine		145 (15.5)	97 (13.3)
	Propofol		240 (25.7)	211 (28.9)
	Etomidate		504 (54.0)	402 (55.1)
	Opioids		335 (35.9)	267 (36.6))
	Benzodiazepines		299 (32.0)	241 (33.1)
	Paralytic: depolarizing		258 (27.6)	214 (29.4)
	Paralytic: non-depolarizing		394 (42.2)	306 (42.0)
	Direct laryngoscopy		414 (44.3)	313 (42.9)
	Video laryngoscopy		501 (53.6)	401 (55.0)
	Fiberoptic		22 (2.4)	18 (2.5)
	Tidal volume (ml), mean ± SD		443±78	442±77
	Positive end-expiratory pressure (mmHg), mean ± SD		6.51±2.61	6.46±2.58

*Data were available for > 98% of patients for all characteristics except SpO_2_, fluid balance, APACHE II score and lactate which were missing for 6%, 12%, 16% and 29% of patients respectively in the Full Cohort, and 6%, 14%, 18% and 35% of patients respectively in the Stable Cohort.

APACHE: acute physiologic and chronic health evaluation; RBC: red blood cell; SD: standard deviation; IQR: interquartile range; CRNA: certified registered nurse anesthetist; NP: nurse practitioner; PA: physician assistant; RRT: registered respiratory therapist

### PIH prediction in the full and stable cohorts

The primary outcome was experienced in 344 (36.8%) patients of the full cohort and in 216 (29.6%) patients of the stable cohort (see S4 Table for summary of cases experiencing each individual outcome). In our full cohort, 11 predictor variables were independently associated with PIH—APACHE II score, age, sepsis, ETI in setting of cardiac arrest or MAP <65 mmHg or acute respiratory failure, diuretic use 24 hours preceding ETI, catecholamine or phenylephrine use 60 minutes preceding ETI, pre-intubation SBP, and etomidate use during ETI: C-statistic 0.75 (95% CI 0.72, 0.78). A second model (stable cohort) that excluded unstable patients and included the above variables except sepsis and etomidate had a C-statistic of 0.71 (95% CI 0.67, 0.75) (Table 3 –the odds ratio estimates are based on the penalized parameter estimates and there is no corresponding standard error for these estimates, hence no confidence intervals).

**Table 3:** Model results for full cohort and stable cohort.

	Full Cohort*		Stable Cohort^†^	
Predictor	coefficient	OR	coefficient	OR
Intercept	-1.750		-1.649	
APACHE II score, per 1 point increase	0.019	1.019	0.009	1.009
Age, per year increase	0.005	1.005	0.005	1.005
Sepsis	0.028	1.028		
Intubation in setting of respiratory failure	0.095	1.100	0.003	1.003
Intubation in setting of MAP < 65 mmHg	0.342	1.408	0.181	1.198
Intubation in setting of cardiac arrest	0.210	1.234		
Diuretics in prior 24 hours	0.315	1.370	0.224	1.251
Catecholamine 60 minutes prior to intubation	0.517	1.677		
Phenylephrine 60 minutes prior to intubation	0.076	1.079	0.036	1.037
Systolic blood pressure				
≥130 mmHg	0.000	1.000	0.000	1.000
per mmHg below 130	0.020	1.020	0.018	1.018
Etomidate used during intubation	-0.135	0.874		
Model performance				
C-statistic	0.75		0.71	
95% CI	0.72 to 0.78		0.67 to 0.75	

*Full cohort (N = 934) was used to derive the HYpotension Prediction Score (HYPS).

^†^Stable cohort (N = 729) excluded unstable patients (those receiving pre-intubation catecholamine pressors and/or who were intubated in the setting of cardiac arrest). This model was used to derive the stable (s) HYPS.

APACHE: acute physiologic and chronic health evaluation; OR: odds ratio; MAP: mean arterial pressure; CI: confidence interval

In a sensitivity analysis, replacing SBP with MAP did not improve the models’ utility (Full cohort: 0.74 [95% CI 0.71, 0.77]; Stable cohort: 0.70 [95% CI 0.66, 0.75]). Thus, we utilized SBP in the calculation of the HYpotension Prediction Score (HYPS—Full Cohort) and the (s)table HYpotension Prediction Score ([s] HYPS—Stable Cohort). The calibration plots and receiver operating characteristic curves are shown in S5 and S6 Tables for both the full and stable cohorts. Tables 4 and 5 illustrate the point scoring system and risk categorization in the full and stable cohorts [22].

**Table 4:** HYpotension Prediction Score (HYPS) and (s)table HYpotension prediction score [(s)HYPS] and risk categorization.

	Full Cohort* (HYPS)	Stable Cohort^†^ [(s) HYPS]
Predictor	Points	Points
APACHE II score		
≤ 10	0	0
11 to 15	1	0.5
16 to 20	2	1
21 to 25	3	1.5
≥ 26	4	2
Age, years		
≤ 40	0	0
41 to 50	0.5	0.5
51 to 60	1	1
61 to 70	1.5	1.5
71 to 80	2	2
≥ 81	3	3
Sepsis diagnosis		
Yes	1	
No	0	
Intubation setting		
Respiratory Failure	1	0‡
MAP < 65 mmHg	3.5	2
Cardiac arrest	2	†
Others	0	0
Diuretics in prior 24 hours		
Yes	3	2.5
No	0	0
Catecholamine 60 minutes prior to intubation		
Yes	5	§
No	0	
Phenylephrine 60 minutes prior to intubation		
Yes	1	0.5
No	0	0
Systolic blood pressure (mmHg)		
≤ 89	10.5	10.5
90 to 99	7	7
100 to 109	5	5
110 to 119	3	3
120 to 129	1	1
≥ 130	0	0
Etomidate used for intubation		
Yes	0	
No	-1.5	

‡Although respiratory failure as the indication for the intubation had a non-zero coefficient in the predictive model, the magnitude of the coefficient was not large enough to assign non-zero points when creating the predictive score for stable patients.

§Patients with this characteristic are considered unstable and not included in the stable cohort.

**Table 5:** Risk categorization for full and stable HEMAIR cohorts.

			Immediate		
			Hypotension	Logistic regression	
Risk Score	Expected Risk	N	# (%)	OR	(95% C.I.)
HYPS-score (Full Cohort)*					
≤ 1.5	Low (≤ 19%)	101	12 (12%)	1.0	Reference
2 to 10.5	Moderate (20–39%)	526	140 (27%)	2.7	(1.4, 5.1)
11 to 18.5	High (40–59%)	211	123 (58%)	10.4	(5.3, 20.1)
≥ 19	Very High (≥ 60%)	96	69 (72%)	19.0	(9.0, 40.1)
(s) HYPS-score (Stable cohort)^†^					
≤ 1	Low (≤ 19%)	81	9 (11%)	1.0	Reference
1.5 to 11.5	Moderate (20–39%)	579	161 (28%)	3.1	(1.5, 6.3)
≥ 12	High (≥ 40%)	69	46 (67%)	16.0	(6.8, 37.6)

*For the full cohort (N = 934), HYPS ranged from -1.5 to 29 (median 7.5, interquartile range 4 to 12.5).

^†^For the stable cohort (N = 729), (s) HYPS ranged from 0 to 18.5 (median 4.5, interquartile range 2.5 to 8).

HYPS: HYpotension Prediction Score; (s)HYPS: (s)table HYpotension Prediction Score; APACHE: acute physiologic and chronic health evaluation; HEMAIR: HEModynamic and AIRway; OR: odds ratio; CI: confidence interval.

### Clinical utility of HYPS and (s) HYPS

The potential bedside clinical utility of HYPS and (s) HYPS are shown in Tables 4 and 5 and S7. The positive predictive value was 11.9% for HYPS and 11.1% for (s) HYPS while the negative predictive value was 88.1% for HYPS and 88.9% for (s) HYPS at the lowest risk threshold. For the highest risk threshold, the positive predictive value was 71.9% for HYPS and 66.7% for (s) HYPS while the negative predictive value was 28.1% for HYPS and 33.3% for (s) HYPS.

## Discussion

The HEMAIR multicenter study retrieved data on ETIs performed in the critically ill to derive and validate a predictive model for immediate hypotension following ETI. We identified 11 variables (increasing APACHE II [per one point], increasing age [per year], sepsis diagnosis, ETI performed in the setting of cardiac arrest or MAP <65 mmHg or acute respiratory failure, use of diuretics 24 hours prior to ETI, use of catecholamines or phenylephrine immediately prior to ETI, decreasing SBP from 130 mmHg [per mmHg], and use of etomidate sedation for ETI) that were independently associated with the primary outcome. Of these 11 variables, etomidate use was found to lower the risk of PIH. We combined these predictors into a risk scoring system that we named HYpotension Prediction Score (HYPS) and stable (s) HYPS to stratify patients’ risk for PIH in both all-comers and stable patients. Both the HYPS and (s) HYPS were acceptable with a validation cohort C-statistic higher than 0.70 with stable calibration plots [23].

Post-intubation hypotension is common in the ICU patient with reported incidences ranging between 20–52% [6, 7, 20, 21]. We report a similar experience with an incidence ranging from 29% to 36%. Presence of PIH, even if limited in duration, is associated with significant morbidity and mortality in the ICU [6]. In the emergency department, PIH has been associated with increased mortality and length of stay [8, 24]. The post-intubation period is one of particular vulnerability to hypotension and nearly a third of all hypotension in the intraoperative period occurs after ETI, with an associated independent and increased risk for postoperative AKI [25].

Age, ETI for acute respiratory failure, pre-intubation hypotension, APACHE, history of obstructive lung disease and renal disease are variables that have been implicated in the pathway to PIH [6–8, 20, 21, 26, 27]. Our data identified some new variables that were not previously associated with immediate hypotension in this setting. For example, we found that not only ETI in the setting of acute respiratory failure and MAP <65 mmHg increases the risk for PIH, but ETI in the setting of cardiac arrest also increases the risk. In our study, patients who suffered a cardiac arrest but had a perfusing rhythm at the time of ETI were included. Peri-cardiac arrest hypotension due to myocardial dysfunction is common and leads to poor outcomes [28, 29]. In addition, data from Get-With-The-Guidelines registry has demonstrated that hypotension surrounding an acute respiratory compromise event is frequently associated with cardiac arrest [30]. We found that diuretic use increases risk of PIH. Sedation and the physiologic effects of positive pressure ventilation commonly results in blood pressure reduction surrounding ETI [9]. Therefore, hypovolemia due to diuresis would plausibly exacerbate blood pressure decreases. Another possibility, although less likely based on Table 1, is that patients who were exposed to diuretics may have been more likely to have congestive heart failure and heart failure itself may well explain the increased prevalence of PIH. Interestingly, the use of etomidate during ETI was protective as compared to other agents. Etomidate does not inhibit sympathetic tone or myocardial function and thus produces minimal hemodynamic changes during ETI; however, this is not without potential harm as etomidate is known to cause adrenal insufficiency and possibly multiorgan failure [11, 31]. Although we noted a protective effect from etomidate, this may have been related to some unmeasured variable (non-randomized design) and therefore systematic bias may have been present. To have a score that is clinically useful in patients not *in extremis*, we excluded those who were receiving pre-intubation catecholamine vasopressors and/or who were intubated in the setting of cardiac arrest ([s] HYPS). We found similar risk factors in this group of patients as in the full cohort with the exception of etomidate use and sepsis.

Traditional blood pressure thresholds of 65 mmHg in the ICU have recently been questioned. A cohort of nearly 9,000 patients demonstrated that the earliest association of myocardial injury, AKI, and mortality occurred at a MAP of 85 mmHg. For mortality and AKI, this harm increased in a progressive manner down to a MAP of 55 mmHg [1]. Our results are consistent with the need for an elevated MAP threshold in the critically ill. We found an increased risk for PIH once SBP fell below 130 mmHg. A SBP of 130 mmHg, even if the diastolic is 2x below normal (40), would correlate to a minimum MAP of 70 mmHg. Interestingly, when MAP was used rather than SBP, we found that a threshold below 95 mmHg was associated with PIH. Thus, perhaps aiming for a higher perfusion pressure in the critically ill, either via MAP or SBP would prevent PIH and associated poor outcomes [32]. This would need to be tested via a future interventional trial.

Our study has several strengths. We enrolled a clinically diverse set of patients from around the country representing 7 regions of the United States. Based on our sample-size analysis, we had a robust sample for our primary outcome. Second, data were collected prospectively and in real-time, allowing for bedside validation of documented blood pressure both before and after ETI. Third, we present a novel score, called the HYpotension Prediction Score (HYPS), that is also clinically useful in more stable patients (stable [s] HYPS) and therefore, may aid the clinician in quickly and efficiently predicting immediate hypotension following ETI. This, in theory, would allow for preemptive adjustment of treatment plans to avoid this immediate complication. For example, implementation of an ETI bundle consisting of fluid loading in the setting of diuretics, choice of etomidate for sedation, and early use of vasopressors with decreasing SBP may reduce immediate hypotension following ETI as demonstrated in one study [33]. Finally, Lee et al. alluded to distinct discriminatory patterns in hemodynamic data that could indicate impending hypotension and called for a hypotensive risk stratifier in the ICU [34]. Considering that even a few minutes of hypotension may be associated with significant risk in this vulnerable population, these easy to use bedside risk scores may help avert preventable harm [2].

### Limitations

First, we did not capture ETIs outside the ICU and thus our results may not be generalizable to non-ICU settings. These patients may be even more vulnerable to untoward physiologic outcomes than the ICU population (and certainly further afield from rescue with vasopressors). However, our intent was to develop a scoring system that would be beneficial in the most severely ill patients, i.e., the critically ill. Second, the providers performing ETI were aware of the study and may have taken precautions to limit complications (no formal protocol was utilized) thereby introducing bias. Third, we had a large amount of missing data for some variables such as APACHE and lactate. However, we used multiple imputations when appropriate and with data from same HHS region. Fourth, although we used LASSO regression with the penalty parameter (λ) chosen using 10-fold cross-validation, we did not validate our model using an external dataset. Fifth, we may not have captured every ICU ETI. Nonetheless, our study did not exclude any ICU ETI and thus, likely represented random sampling. Sixth, there may be multiple other variables related to PIH not included in the analysis. Our dataset was fairly extensive and thus we feel the majority of variables related to PIH were captured. Finally, the upper risk cut-off could have been extended. However, we chose the upper risk cut-off in both cohorts based on the reasoning that differentiating those at lower risk was felt to be more relevant than those at higher risk as most clinicians would have likely altered their plans if the risk for PIH was already high.

## Conclusions

The HYPS and (s) HYPS are practical, validated tools that can be calculated using clinically available information. These scores effectively identify those individuals who are at increased risk for PIH in all-comers and in those not *in extremis*. The utility of both scores in the ICU—including its additive efficacy compared with unassisted clinical decision making—requires further research.

## Supporting information

S1 Table HEMAIR collaborators. HEMAIR: HEModynamic and AIRway collaborators, HHS: Health & Human Services.
(DOCX)

S2 Table Candidate predictor variables. (DOCX)

S3 Table Data analysis centers. HHS: Health & Human Services.
(DOCX)

S4 Table Summary of cases experiencing each individual outcome (0 = no, 1 = yes). SBP: systolic blood pressure, MAP: mean arterial pressure.
(DOCX)

S5 Table Calibration plots (Patients grouped according to deciles of predicted risk score. The observed rate is plotted against the mean predicted risk score for the given decile. For the lowest decile, the mean score was ~20%, but there were individuals with scores below this.).
(DOCX)

S6 Table Receiver Operating Characteristic (ROC) curves. (DOCX)

S7 Table Predicted risk of post-intubation hypotension by risk score. Reference lines separating the 4 risk categories in HYpotension Prediction Score, Reference lines separating the 3 risk categories in (s)table HYpotension Prediction Score.
(DOCX)

S8 Table Minimal underlying data set. (CSV)
