# Supplementary material for: Risk factors for and prediction of post-intubation hypotension in critically ill adults: A multicenter prospective cohort study
Source: PLoS One. 2020 Aug 31;15(8):e0233852. doi: 10.1371/journal.pone.0233852 (PMC7458292; doi:10.1371/journal.pone.0233852)
Supplement: S1 Table — HEMAIR: HEModynamic and AIRway collaborators, HHS: Health & Human Services. (DOCX) [file pone.0233852.s001.DOCX]

**S1 Table. HEMAIR Collaborators.**

HHS region 1:

**Berkshire Medical Center** - Cynthia Callahan, Esteben Delpilar, Leslie Drager, Tammy Bator

**Bridgeport Hospital** - David Kaufman, Jasleen Pannu

HHS region 3:

**Geisinger Health System** - Uchenna Ofoma, Brittany Burke

HHS region 4:

**Mayo Clinic, Jacksonville** - Pablo Moreno Franco, Vikas Bansal, Karen Hampton

**University of Kentucky** - Ashley Montgomery-Yates, Peter Morris, Evan P. Cassidy

HHS region 5:

**Aurora Healthcare** - Ernesto Brauer; David Kramer, Carol Halliday, Carol Tutino

**Cleveland Clinic** - Ashish Khanna, Mariya Geube, Amanda J. Naylor, Eric M. Reville, Quinton M. Riter

**Detroit Medical Center** - Sarah Lee

**Mayo Clinic, Rochester -** Nathan Smischney, Rahul Kashyap, Mohamed Seisa, Daniel Diedrich, Darrell R. Schroeder, Timothy Weister, Mukesh Kumar, Daniel Brown, Philippe Bauer, Ognjen Gajic

HHS region 6:

**Corpus Christi Medical Center** - Salim Surani, Abhay Vakil, Bryan Anderson

**University of Oklahoma Health Sciences Center** - Gozde Demiralp, Ashley Bailey-Classen, Amir Butt

HHS region 7:

**Creighton University** - Lee E. Morrow; Mary T. Pote, Theresa Ramelb

**Mercy Hospital** - Chakradhar Venkata, Jackie O’Brien

HHS region 9:

**Mayo Clinic, Scottsdale** - Ayan Sen, Pritee Tarwade

**Memorial Medical Center** - Rudy Tedja, Karen L. Swanson, Ann Axford, Brenda Lynch

**University of South California** - Santhi Kumar, Mark Slootsky, James Moore

HEMAIR: HEModynamic and AIRway collaborators, HHS: Health & Human Services.
