# Supplementary material for: Risk factors for and prediction of post-intubation hypotension in critically ill adults: A multicenter prospective cohort study
Source: PLoS One. 2020 Aug 31;15(8):e0233852. doi: 10.1371/journal.pone.0233852 (PMC7458292; doi:10.1371/journal.pone.0233852)
Supplement: S2 Table — (DOCX) [file pone.0233852.s002.DOCX]

**S2 Table. Candidate Predictor Variables.**

*Patient Characteristics*

1. Age
2. Body mass index
3. Acute Physiologic And Chronic Health Evaluation II
4. Congestive heart failure
5. Coronary artery disease
6. Obstructive lung disease
7. End-stage renal disease
8. Cirrhosis
9. Diabetes mellitus II

*Care Process Characteristics (Pre-intubation)*

1. Renal replacement therapy
2. Hypovolemic shock
3. Sepsis
4. Mechanical circulatory support
5. Cardiovascular medications
6. Vasoactive medications
7. Total fluid balance
8. Sedative medications
9. Fluid bolus
10. Non-invasive ventilation or high-flow nasal cannula
11. Tracheal intubation timing
12. Intubation setting/indication
13. Hypoxemia
14. Systolic blood pressure and mean arterial pressure
15. Shock and modified shock index

*Laboratory Characteristics (Pre-intubation)*

1. Lactate level
2. Hemoglobin level

*Care Process Characteristics (Peri-intubation)*

1. Intubation medications

*Provider Characteristics*

1. Training level
2. Intubation attempts
