# Supplementary material for: Risk factors for and prediction of post-intubation hypotension in critically ill adults: A multicenter prospective cohort study
Source: PLoS One. 2020 Aug 31;15(8):e0233852. doi: 10.1371/journal.pone.0233852 (PMC7458292; doi:10.1371/journal.pone.0233852)
Supplement: S3 Table — HHS: Health & Human Services. (DOCX) [file pone.0233852.s003.DOCX]

**S3 Table. Data Analysis Centers.**

| **No.** | **HHS Region** | **Medical Center (n)** | **City** | **State** |
| --- | --- | --- | --- | --- |
| 1 | 5 | Aurora Healthcare (126) | Milwaukee | Wisconsin |
| 2 | 1 | Berkshire Medical Center (41) | Pittsfield | Massachusetts |
| 3 | 1 | Bridgeport Hospital/Yale (64) | Bridgeport | Connecticut |
| 4 | 5 | Cleveland Clinic (121) | Cleveland | Ohio |
| 5 | 7 | Creighton University (50) | Omaha | Nebraska |
| 6 | 6 | Corpus Christi Medical Center (24) | Corpus Christi | Texas |
| 7 | 5 | Detroit Medical Center / Wayne State University (3) | Detroit | Michigan |
| 8 | 3 | Geisinger Health System (70) | Danville | Pennsylvania |
| 9 | 4 | Mayo Clinic, Jacksonville (61) | Jacksonville | Florida |
| 10 | 5 | Mayo Clinic, Rochester (79) | Rochester | Minnesota |
| 11 | 9 | Mayo Clinic, Scottsdale (59) | Scottsdale | Arizona |
| 12 | 9 | Memorial Medical Center (45) | Modesto | California |
| 13 | 7 | Mercy Hospital (63) | Saint Louis | Missouri |
| 14 | 4 | University of Kentucky (49) | Lexington | Kentucky |
| 15 | 6 | University of Oklahoma Health  Sciences Center (52) | Oklahoma City | Oklahoma |
| 16 | 9 | University of South California (27) | Los Angeles | California |

HHS: Health & Human Services
