# Supplementary material for: Risk factors for and prediction of post-intubation hypotension in critically ill adults: A multicenter prospective cohort study
Source: PLoS One. 2020 Aug 31;15(8):e0233852. doi: 10.1371/journal.pone.0233852 (PMC7458292; doi:10.1371/journal.pone.0233852)
Supplement: S4 Table — SBP: systolic blood pressure, MAP: mean arterial pressure. (DOCX) [file pone.0233852.s004.DOCX]

**S4 Table. Summary of cases experiencing each individual outcome (0=no, 1=yes).**

Full Cohort

| SBP <80 mmHg  or 40% drop | MAP <65 mmHg | Initiation or increase vasopressor | Number of cases |
| --- | --- | --- | --- |
| 0 | 0 | 1 | 149 |
| 0 | 1 | 0 | 44 |
| 0 | 1 | 1 | 41 |
| 1 | 0 | 0 | 10 |
| 1 | 0 | 1 | 6 |
| 1 | 1 | 0 | 38 |
| 1 | 1 | 1 | 56 |

SBP: systolic blood pressure; MAP: mean arterial pressure

Stable Cohort

| SBP <80 mmHg or 40% drop | MAP <65 mmHg | Initiation or increase vasopressor | Number of cases |
| --- | --- | --- | --- |
| 0 | 0 | 1 | 75 |
| 0 | 1 | 0 | 36 |
| 0 | 1 | 1 | 26 |
| 1 | 0 | 0 | 10 |
| 1 | 0 | 1 | 4 |
| 1 | 1 | 0 | 32 |
| 1 | 1 | 1 | 33 |

SBP: systolic blood pressure; MAP: mean arterial pressure
