# Supplementary material for: Risk factors for and prediction of post-intubation hypotension in critically ill adults: A multicenter prospective cohort study
Source: PLoS One. 2020 Aug 31;15(8):e0233852. doi: 10.1371/journal.pone.0233852 (PMC7458292; doi:10.1371/journal.pone.0233852)

**S5 Table. Calibration Plots (Patients grouped according to deciles of predicted risk score. The observed rate is plotted against the mean predicted risk score for the given decile. For the lowest decile, the mean score was ~20%, but there were individuals with scores below this.).**

Full Cohort


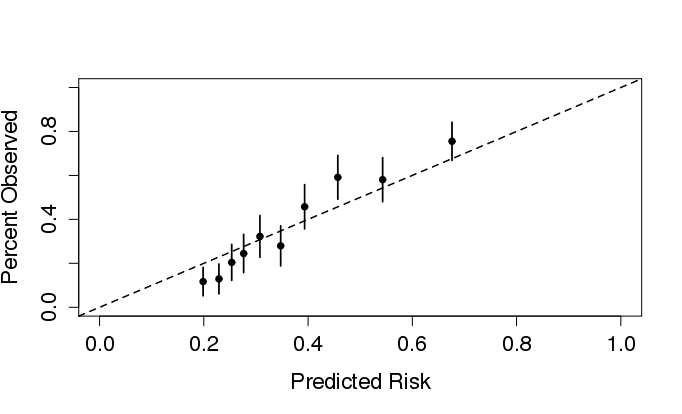


Stable Cohort


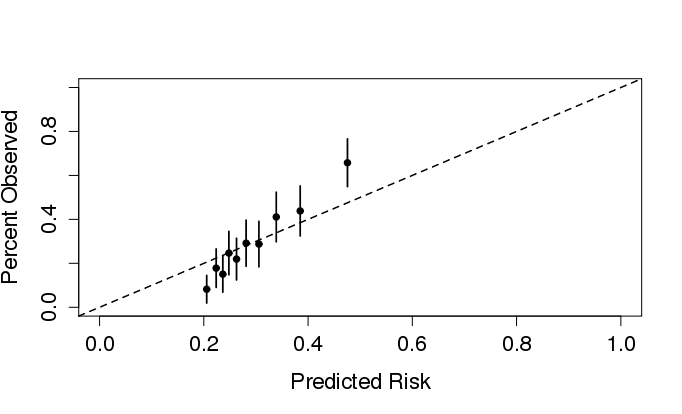

Supplement: S5 Table — The observed rate is plotted against the mean predicted risk score for the given decile. For the lowest decile, the mean score was ~20%, but there were individuals with scores below this.). (DOCX) [file pone.0233852.s005.DOCX]
