# Supplementary figures and images for: Risk factors for and prediction of post-intubation hypotension in critically ill adults: A multicenter prospective cohort study
Source: PLoS One. 2020 Aug 31;15(8):e0233852. doi: 10.1371/journal.pone.0233852 (PMC7458292; doi:10.1371/journal.pone.0233852)

**S6 Table. Receiver Operating Characteristic (ROC) Curves.**

Full Cohort


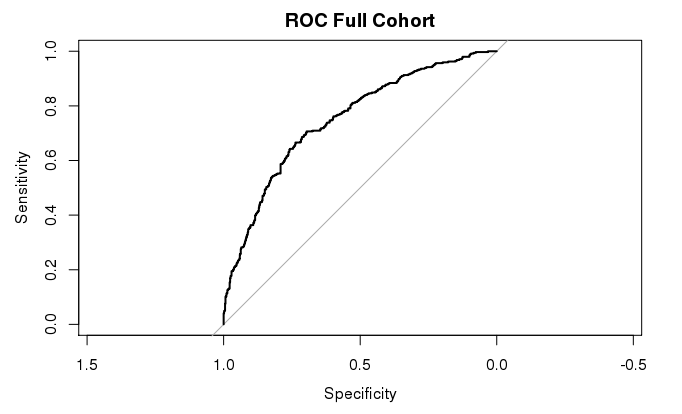


Stable Cohort


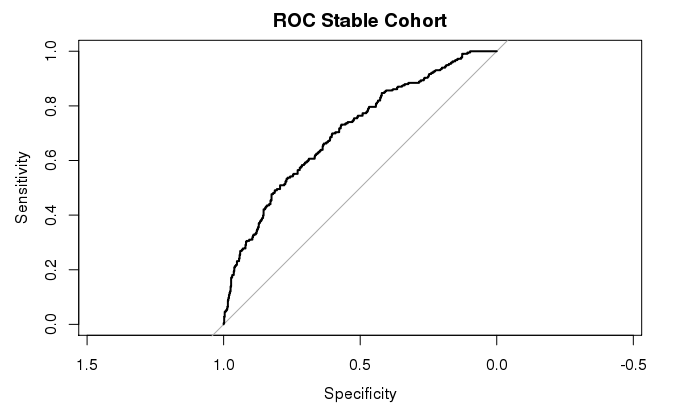

Supplement: S6 Table — (DOCX) [file pone.0233852.s006.DOCX]
