# Supplementary material for: Risk factors for and prediction of post-intubation hypotension in critically ill adults: A multicenter prospective cohort study
Source: PLoS One. 2020 Aug 31;15(8):e0233852. doi: 10.1371/journal.pone.0233852 (PMC7458292; doi:10.1371/journal.pone.0233852)
Supplement: S7 Table — Reference lines separating the 4 risk categories in HYpotension Prediction Score, Reference lines separating the 3 risk categories in (s)table HYpotension Prediction Score. (DOCX) [file pone.0233852.s007.DOCX]

**S7 Table. Predicted Risk of Post-Intubation Hypotension by Risk Score.**


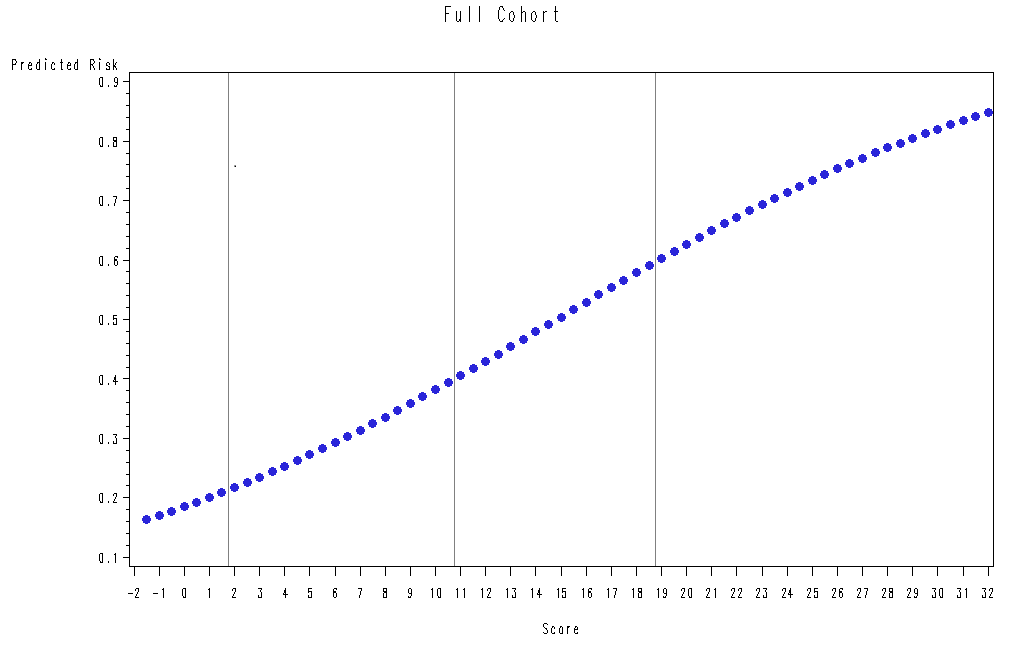


Reference lines separating the 4 risk categories in HYpotension Prediction Score.


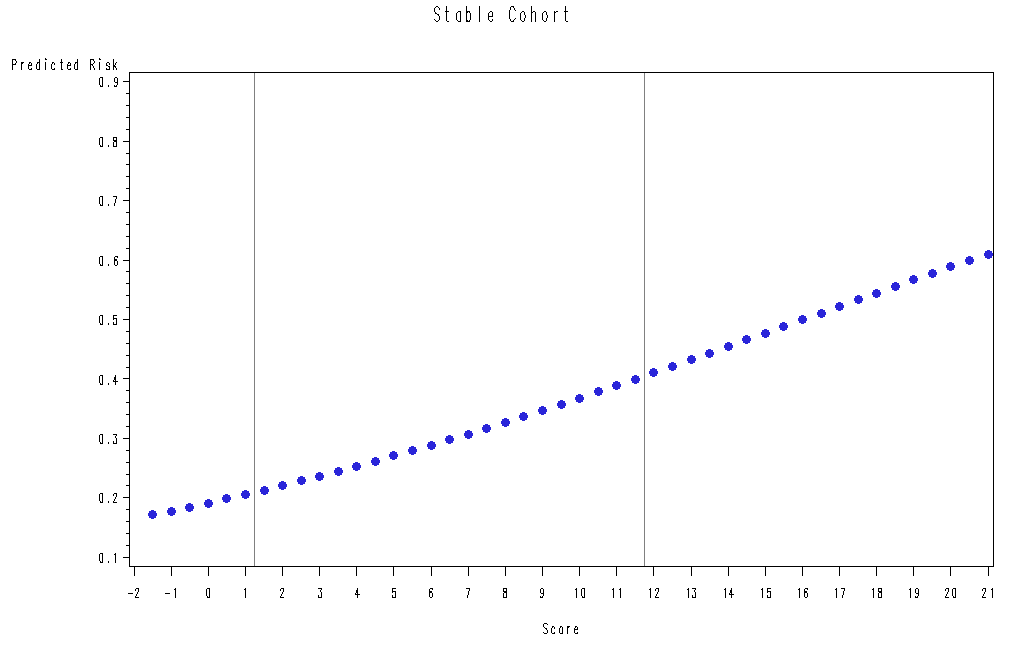


Reference lines separating the 3 risk categories in (s)table HYpotension Prediction Score.
